# Genetic and Phenotypic Characteristics of Five *Staphylococcus aureus* Strains Isolated from Yakutian Cattle

**DOI:** 10.3390/ani16081189

**Published:** 2026-04-14

**Authors:** Ksenia Fursova, Daria Nikanova, Sergei Sokolov, Daria Sherman, Olga Artem’eva, Evgenia Kolodina, Anna Tiurina, Anatoly Sorokin, Timur Dzhelyadin, Varvara Romanova, Margarita Shchannikova, Andrei Pochtovyi, Vladimir Gushchin, Artem Ermakov, Natalia Zinovieva, Fedor Brovko

**Affiliations:** 1 Laboratory of Immunochemistry, Shemyakin and Ovchinnikov Institute of Bioorganic Chemistry of the Russian Academy of Sciences, 142290 Pushchino, Russia; 2 Laboratory of Microbiology, L.K. Ernst Federal Science Center for Animal Husbandry, 142132 Dubrovitsy, Russia; dap2189@gmail.com (D.N.);; 3 Laboratory of Plasmid Biology, G.K. Skryabin’ Institute of Biochemistry and Physiology of Microorganisms, Federal Research Center “Pushchino Scientific Center for Biological Researches”, Russian Academy of Sciences, 142290 Pushchino, Russia; 4 Lopukhin Federal Research and Clinical Center of Physical-Chemical Medicine of Federal Medical Biological Agency, 119435 Moscow, Russia; 5 Faculty of Biotechnology, Lomonosov Moscow State University, 119991 Moscow, Russia; 6 Institute of Cell Biophysics of the Russian Academy of Sciences Russia, 142290 Pushchino, Russia; 7 Laboratory of Selection and Breeding of Cattle, M.G. Safronov Yakut Scientific Research Institute of Agriculture, 677001 Yakutsk, Russia; varvara.romanova.59@mail.ru; 8 Federal State Budget Institution “National Research Center for Epidemiology and Microbiology Named after the Honorary Academician N.F. Gamaleya” of the Ministry of Health of the Russian Federation, 123098 Moscow, Russia; 9 Department of Medical Genetics and Postgenomic Technologies, Sechenov First Moscow State Medical University, 119991 Moscow, Russia; 10 Department of Virology, Biological Faculty, Lomonosov Moscow State University, 119991 Moscow, Russia; 11 Institute of Theoretical and Experimental Biophysics, Russian Academy of Sciences, 142290 Pushchino, Russia; 12 Nanoporus LLC, 142200 Serpukhov, Russia

**Keywords:** *S. aureus*, mastitis, Yakutian cattle, virulence genes, exotoxins, cytotoxins, adhesins, antibiotic resistance, hemolysins

## Abstract

To date, the problem of mastitis in cattle remains relevant for both the industrial sector and scientific research. Despite numerous active investigations, the causes of this disease have not been fully established. It is postulated that several factors may be involved, such as bacterial pathogens, animal husbandry practices, and weather and climatic conditions. In this study, we selected cows from farms in Yakutia to investigate microbial isolates present in the milk of cows affected by mastitis and treated with antibiotics. Five identified *Staphylococcus aureus* isolates were investigated using whole-genome sequencing (Illumina sequencing and nanopore sequencing), followed by analysis of virulence factors in the genomes and cultural properties of the isolates. The profile of *S. aureus* virulence genes (exotoxins, cytotoxins, superantigen-like proteins, adhesins) was identified via WGS. Hemolysin gene (*hla*) was detected in all isolates. An investigation of the cultural properties of the isolates, specifically through hemolysis of rabbit erythrocytes and Western blot analysis of the culture liquid of *S. aureus*, revealed different expression levels of alpha-hemolysin among the strains. One isolate (17-21) exhibited the highest secretion level of about 320 ± 37 ng, both in the hemolysis test and immunoblotting assay. An investigation of the isolates’ antibiotic resistance showed that all isolates exhibited multidrug resistance, as confirmed by the presence of antibiotic resistance genes in these isolates. One isolate (7-7) exhibited the broadest range of phenotypic resistance and was resistant to all tested antibiotics (except clindamycin). Phylogenetic analysis suggested that the evolution of these isolates occurred independently in their respective ecological niches, although their transfer from cattle to humans, and vice versa, is possible. Isolates 7-7, 18-22, 33-40, and 35-42 are most typical to Yakutian cattle, while isolate 17-21 might have been introduced from a different region. To the best of our knowledge, this is the first in-depth study into a range of *S. aureus* isolates associated with mastitis infection in Yakutian cattle.

## Simple Summary

In this study, we investigated the genetic and phenotypic characteristics of five *Staphylococcus aureus* strains isolated from milk samples of Yakutian cows. An analysis of the complete genome sequences with subsequent annotation allowed us to determine the profile of virulence genes (exotoxins, cytotoxins, superantigen-like proteins, adhesins) of *S. aureus* strains isolated from cow milk. All isolates were found to exhibit multidrug resistance, confirmed by the presence of antibiotic resistance genes in these isolates. This is the first in-depth study of a range of *S. aureus* isolates associated with mastitis in Yakutian cows.

## 1. Introduction

Bovine mastitis is a significant problem causing substantial economic damage in the dairy industry [1]. Among the various causes of mastitis, dysbiosis plays a major role [2,3,4,5], with streptococci, *Staphylococcus aureus*, and *Escherichia coli* acting as the most common pathogens [6,7,8,9]. The geographical and climatic patterns of the distribution of individual pathogens are of great importance [10,11,12,13].

The Yakutian cattle (Bos Taurus Turano-mongolicus) is an indigenous breed developed through many years of traditional selection. It is one of the endangered breeds of farm animals. It is highly resistant to various infectious diseases, including mastitis. In this study, we investigated microbial isolates obtained from the milk of Yakutian cows.

Yakutia is the largest region in the Russian North. Yakutia’s location in the northeast of the vast Eurasian continent means an extremely continental climate, with temperatures fluctuating sharply between seasons. In the natural climate zoning system, Yakutia is classified as extremely harsh regarding temperature and exceptionally arid regarding precipitation. Of all the environmental factors, low air temperature is the most important factor while simultaneously the least stable. Temperatures in the winter months drop to −60–65 °C, while in the summer, they rise to 33–38 °C. Most of the territory is in the middle taiga zone, which transitions to forest–tundra and tundra zones to the north. Yakutia has a unique climate, and a key climate feature for livestock farming is the low temperatures generated by permafrost.

Yakutia is a distinct and large region with its own unique agricultural practices, which have no analogues in the country. Traditional agricultural sectors include reindeer herding, livestock farming, horse breeding, hunting, and fishing. The diet of Yakutian cattle consists mainly of grass in the warm season and hay with minor additions of mixed feed in the winter period. With the onset of cold weather, the udder becomes covered with thick hair, which reliably protects the small teats from the cold. Yakut cows usually self-wean during this period, an adaptation to the harsh conditions. Consequently, their lactation period is shorter than that of other breeds. Yakut cows have low milk productivity, ranging from 778 to 1673 kg/year, but the fat content in the milk reaches 8.5%. Mastitis in Yakutian cows is mostly caused by improper milking machines, violation of the milking regime, mechanical damage to the udder in closed farm buildings during the winter, hypothermia, and damage to the udder due to insect bites.

The goal of this study was to study the genomic and functional characteristics of *S. aureus* strains isolated from the milk of Yakutian cows that had suffered a single episode of mastitis and received a course of antibiotic therapy.

Milk samples were collected from farms in four districts (uluses) of Central Yakutia (Ust-Aldansky, Megino-Kangalassky, Khangalassky, and Suntarsky districts). This area is the most favorable zone for the development of agriculture. We selected farms where no crossbreeding with other breeds had been performed. The animals of these farms had well-characterized pedigrees, and their external characteristics demonstrated affiliation with the regional breed. Forty-six milk samples were collected from cows that had recovered from mastitis and were treated with antibiotics. At the time of collection, the cows were considered healthy. Using microbiological techniques, we isolated 21 pure CNS cultures and 5 pure *S. aureus* cultures, whose genetic and phenotypic characteristics are presented in this article.

The study materials were collected from remote Yakutian farms nearly isolated from the outside world, so we could determine the evolutionary characteristics of the obtained microbial strains and their pathogenicity factors. Given that the cows are in close contact with the farm personnel in a confined space, it is highly likely that microorganisms are regularly transferred from animals to humans, and vice versa. Therefore, this study investigated the comparative homology (variability) of the genomes and individual genes of staphylococci isolated from Yakut cows and those of cows and human patients deposited in GenBank.

## 2. Materials and Methods

### 2.1. Sample Collection and Preparation

Forty-six milk samples were collected from Yakut cows (Bos Taurus Turano-mongolicus) from four farms. In this study, we aimed to evaluate the condition of animals that had successfully recovered from a single episode of mastitis. Treatment was carried out with antibiotic therapy. For this purpose, health assessments were conducted using veterinary medicine methods. The general condition of each animal was assessed using KENOTEST (RABOS Intl. Ltd., Moscow, Russia), and an examination of the mammary gland and inguinal lymph nodes was performed. Detection of somatic cells in the milk samples was conducted using a Somatos Mini analyzer (SibAgroTechniques, Novosibirsk, Russia). Animals with a somatic cell count in the range of 90–120 thousand were considered healthy, while those with a somatic cell count over 140 thousand were considered sick.

To perform bacteriological testing for mastitis, milk samples were collected from udder quarters that responded to the KENOTEST and yielded a positive settling test (after 16 h of cooling, milk collected from the affected quarter of the udder formed flakes or clots due to the elevated somatic cell count, indicating latent mastitis). For confirmation, the samples were subjected to somatic cell count using a Somatos Mini analyzer (SibAgroTechniques, Russia). Microbiological testing was conducted according to the “Guidelines for the Bacteriological Testing of Milk and Udder Secretions of Cows” (Minsk, 2008).

The animals were treated with a combination of antibiotics, comprising penicillin, streptomycin, and erythromycin. The effectiveness of the antibiotic treatment was assessed via palpation of the mammary glands and by considering the number of somatic cells in the milk samples. Two months after the end of treatment, milk samples were collected to isolate microorganisms. The milk samples were collected after thorough sanitization of the udder with a disinfectant solution, and any remaining moisture was removed with a sterile napkin. For each sample, 10 mL of milk was collected in a sterile 50 mL Falkon tube (BD, Franklin Lakes, NJ, USA). The samples were transported to the laboratory at a temperature of +4 °C within two hours or frozen at −20 °C.

### 2.2. Isolation of S. aureus

Salt Meat Broth (HiMedia Laboratories Pvt., Ltd., Maharashtra, India) was inoculated with the milk samples at a ratio of 1:9, and the mixture was kept at 37 °C for 18–24 h. Internationally recognized traditional phenotypic methods were applied to characterize bacterial isolates, including Gram-stained colony microscopy, growth on Baird–Parker Agar (HiMedia Laboratories Pvt., Ltd., India), hemolysis on Azide Blood Agar (Pronadisa, Conda, Madrid, Spain), positive result upon plasma coagulation, and biochemical identification. The isolated strains were stored in Trypticase Soy Broth (TSB) (Merck, Darmstadt, Germany) with 30% sterile glycerin at −18 °C.

### 2.3. DNA Extraction

*S. aureus* isolates were cultivated in TSB at 37 °C on an orbital shaker for 14–16 h. The culture liquid was centrifuged at 4000× *g* for 5 min at 4 °C, and the cell pellet was used for DNA extraction.

Five isolates were selected for whole-genome sequencing. DNA samples were extracted using the lysostaphin and GenElute bacterial genomic DNA kit (Sigma-Aldrich, Merck, Darmstadt, Germany). The extracted DNA was analyzed by electrophoresis in a 1% agarose gel stained with ethidium bromide (5 μg/mL).

### 2.4. Genome Sequencing and Data Preprocessing

#### 2.4.1. Illumina Sequencing

Sequencing was performed using an Illumina Inc. system (San Diego, CA, USA) with MiSeq Reagent Kit v3 chemicals. The quality of the sequencing results was assessed with FastQC, and the sequences were trimmed at Bjorn Usadel Lab, Aachen, Germany, to keep the quality above 25.

#### 2.4.2. Nanopore Sequencing

A MinION sequencer with a FLO-MIN106 flow cell (Oxford Nanopore Technologies, Oxford, UK) was used for nanopore sequencing. A ligation sequencing kit SQK-LSK109 (Oxford Nanopore Technologies, Oxford, UK) was used for library preparation. The KAPA HyperPlus kit (KAPA-biosystems, Wilmington, MA, USA) was used for paired-end library preparation. Unicycler 0.5.0 (St. Petersburg, Russia) and Flye 2.9 software were used for hybrid assembly of Illumina and nanopore reads. Bowtie2 version 2.3.5.1 and Pilon version 1.23 were used for nanopore error correction using Illumina data. Circularization of the replicon (chromosome) ends was confirmed by overlapping ends.

### 2.5. Genome Annotation and Deposition

The trimmed reads were uploaded to bv-brc.org [14], and the genomes were assembled and annotated. All the genomes were deposited to GenBank [15].

### 2.6. SNP Phylogeny of Whole-Genome Sequences

The single nucleotide polymorphism (SNP) tree was constructed using CSI Phylogeny 1.4 (Center for Genomic Epidemiology) with the default settings and excluding heterozygous SNPs. Strains were randomly selected from GenBank [15] using the “host name” filter to select strains belonging to a specific host—human or bovine. The following criteria for high-quality SNP calling and filtering were followed: (i) a minimum depth of 10× at SNP positions; (ii) a minimum relative depth of 10% at SNP positions; (iii) a minimum distance of 10 bp between SNPs; (iv) a minimum SNP quality of 30; (v) a minimum read mapping quality of 25; and (vi) a minimum Z score of 1.96. Site validation for each SNP position was performed. SNPs that failed to meet the necessary requirements were excluded from the final analysis. Based on the concatenated alignments of high-quality SNPs, maximum likelihood trees were created using MEGA7 [16].

### 2.7. Multilocus Sequence Typing

SRST2 v0.2.0 [17] software was used to map the trimmed reads to the multilocus sequence typing (MLST) database [18]. The strain definition files for *S. aureus* were downloaded as described in the SRST2 manual, and the analysis was performed with the SRST2 default parameter values.

### 2.8. Virulence Gene Identification

An investigation of the presence of virulence factor genes was conducted using the bv-brc.org server [14]. Only genomes placed close to *S. aureus* on the bv-brc.org phylogenetic analysis tree were used.

### 2.9. Gene Homology

To study gene homology, we used five *Staphylococcus aureus* isolates from Yakut cows and human isolates identified from GenBank. The strain search was conducted using the GenBank database [15]. Authors used the “host name” filter to select strains belonging to a specific host—human or bovine. In addition, several strains obtained from both humans and bovines in various regions of the Russian Federation, including strains with geographical adjacent to Yakutia were selected for analysis.

### 2.10. Antibiotic Resistance Investigation

Antibiotic susceptibility testing was performed using the disc diffusion method for the following antibiotics (HiMedia Laboratories Pvt., Ltd.) on Mueller–Hinton Agar (HiMedia Laboratories Pvt., Ltd.): penicillin, 10 iu; cefoxitin, 30 mkg; ciprofloxacin, 5 mkg; gentamicin, 10 mkg; kanamycin, 30 mkg; erythromycin, 15 mkg; clindamycin, 2 mkg; lincomycin, 15 mkg; rifampicin, 5 mkg; flucidin, 10 mkg; and tetraciclin, 30 mkg. The dishes with inoculated cultures were incubated under aerobic conditions at 37 ± 1 °C for 18–24 h. The *Staphylococcus aureus* (MSSA) ATCC 25923 strain, obtained from the collection of the State Scientific Center for Applied Microbiology and Biotechnology (SSC AMB, Moscow, Russia), was used as a positive control; *Staphylococcus aureus* (MRSA) NCTC 12493 (BD Diagnostics, Franklin Lakes, NJ, USA) was used as a negative control. Sterile saline solution (PanEco, Moscow, Russia) was used as a negative control. Sensitivity was evaluated based on the diameters of the zones of inhibition in accordance with the Performance Standards for Antimicrobial Disc Susceptibility tests, EUCAST [19].

### 2.11. Hemolytic Activity Determination

Assays for hemolytic activity in culture supernatants were performed. *S. aureus* strains were grown at 37 °C in 5 mL TSB without antibiotic selection on an incubator rotary shaker. The samples were collected at 1 h intervals. After centrifugation at 3000× *g* for 10 min to pellet the bacteria, the supernatant was filtered using a 0.22 mkm membrane (Merck Millipore, Tullagreen, Ireland) and diluted, and aliquots were added to a 1% suspension of washed rabbit erythrocytes in 0.01 M phosphate-buffered saline (PBS; pH 7.2) containing 0.1% bovine serum albumin for the hemolysis assay. Serial dilutions of purified toxins in 0.5 mL of diluent (0.01 M sodium phosphate-buffered saline with 0.1% albumin (pH 7.2)) were performed, followed by incubation with 0.5 mL of 1% suspension of washed rabbit erythrocytes at 37 °C for 30 min. The purified hemolysin (95% purity) of *S. aureus* was used as a positive control (kindly provided by Noskov N.A., Gamaleya Research Center Epidemiology and Microbiology, Moscow). Protein A of *S. aureus* (SpA) was used as a negative control. The hemolysis activity was determined by the A560 nM on a Mark immunoplate reader (Biorad Lab, Hercules, CA, USA). The assays were performed twice, and the titer was expressed as the mean of the two values [20]. To remove non-specific binding to protein A, rabbit immunoglobulins were isolated from rabbit plasma through a combination of 40% ammonium sulfate precipitation followed by affinity chromatography on protein A Sepharose (GE Health Care, Chicago, IL, USA), according to the manufacturer’s instructions.

### 2.12. Western Blot Analysis of Culture Liquid of S. aureus

The samples were centrifuged at 5000× *g* for 20 min at 4 °C. The cells were centrifuged, and secreted proteins were precipitated from the cell-free supernatants using 10% TCA. The pellet was suspended using cold ethanol 80% and centrifugated at 12,000× *g* for 5 min twice. The precipitate was washed with aqueous ethanol, dissolved in the sample buffer, and subjected to SDS–PAGE in 10.0% [21] electrophoresis, followed by electrotransfer. Protein electrotransfer to a nitrocellulose membrane was performed in Tris-Ac buffer at pH 7.4 with the addition of 20% ethanol for 15 h at 20 mA. The membrane was stained with 0.1% Ponceau C solution in 0.5 M acetic acid, washed with Milli-Q water twice, and transferred to a blocking solution of 2% BSA/TBST (Tris-Ac, pH 7.4, 0.5% Tween 20, 2% BSA) for 1 h at 25 °C. Then, it was washed with PBST buffer 3 times for 5 min. To block staphylococcal protein A, the membrane was incubated in PBST containing 0.1 mg/mL rabbit antibodies. To detect Hly, the monoclonal antibodies against alpha toxin obtained earlier were used. HRP-conjugated goat anti-mouse IgG (H + L) secondary antibody (Thermo Fisher Scientific, Waltham, MA, USA) and protein A P3838 (Sigma-Aldrich Chemie GmbH, Munich, Germany) were used. Diaminobenzidine (DAB) solution was used for detection. The protein content on the blot was analyzed using a positive control hemolysin band via http://www.gelanalyzer.com. At least three replicates were run, and statistics were calculated using a standard Excel package.

## 3. Results

### 3.1. Phylogenetic Analysis of the Isolates

The results of the MLST analysis and Spa typing of the genomes of strains identified in this study are presented in Table 1. The sequence typing and clonal complexes determination showed that four isolates (7-7, 18-22, 33-40, and 35-42) belonged to ST1651, while one isolate, 17-21, was categorized as type ST97/CC97.

### 3.2. Genome Homology

**Figure 1:**
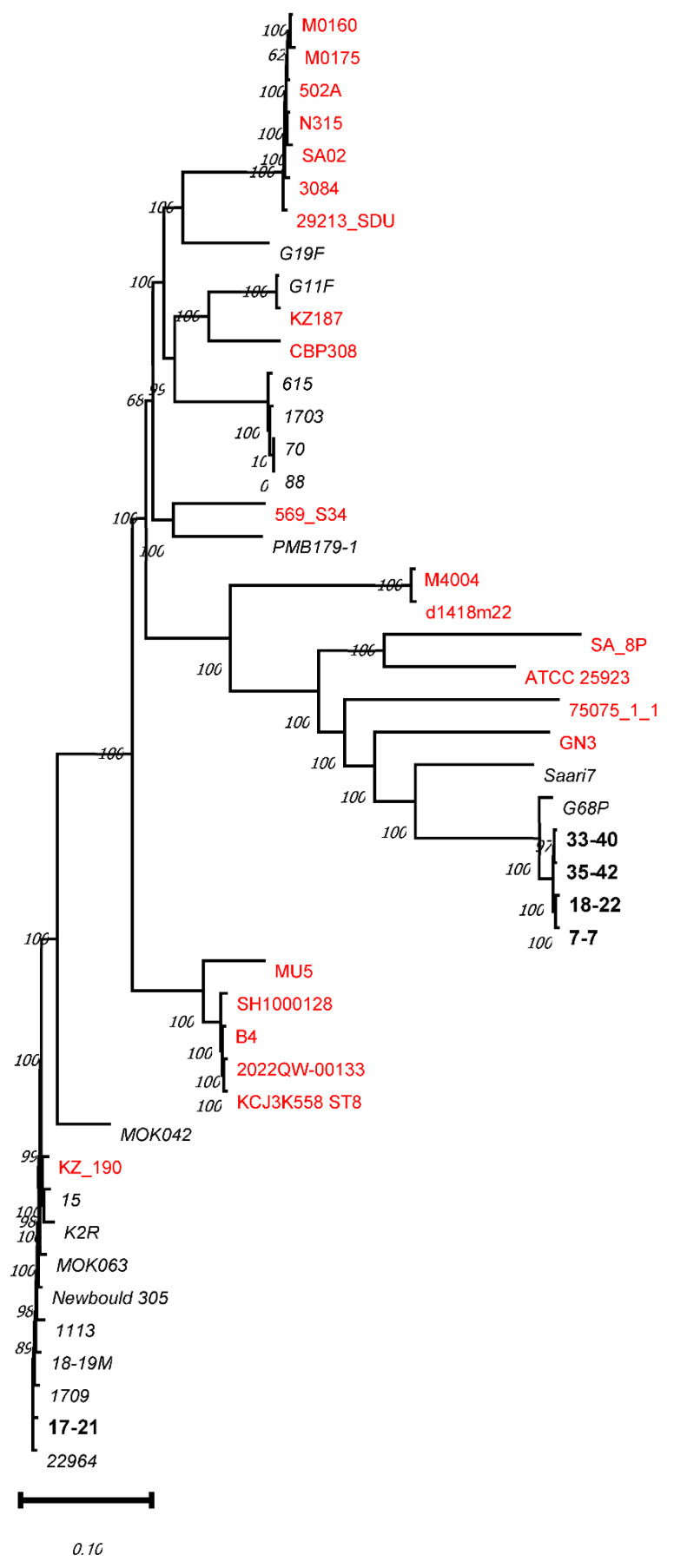
SNP phylogenetic analysis of five *S. aureus* strains isolated from Yakutian cows.

SNP analysis was conducted to examine differences in the isolates’ genomes and determine their potential relationships (Figure 1). Four isolates identified from Yakutian cows, namely, 33-40, 35-42, 18-22 and 7-7, formed a cluster related to the strains G68P and Saari identified in cows, whereas the isolate 17-21 belonged to a distant phylogenetic clade. Phylogenetic analysis of these staphylococcus strains suggested that their evolution occurred independently in their respective ecological niches, although their transfer from cattle to humans, and vice versa, is possible. Isolates 7-7, 18-22, 33-40, and 35-42 are most typical to Yakutian cattle, while isolate 17-21 might have been introduced from a different region.

### 3.3. Pathogenicity Factors

The virulence factors in the five isolates from Yakutian cows were determined. Table 2 shows that these isolates possessed a genetic pool composed of enterotoxins, exotoxins, cytotoxins, pore-forming hemolysins, and leucocidins.

Specifically, four isolates (7-7, 18-22, 33-40, 35-42) possessed a set of enterotoxins (*seo*, *sem*, *sei*, *seu*, *sen*, *seg*), while 17-21 lacked these genes. Enterotoxin-like proteins genes (*ssl*) were detected in all isolates. Genes of the cytotoxins hemolysins (*hla*, *hlg*, *hld*) and leukocidins were detected in all five Yakutian isolates.

### 3.4. Adherence Factors

The genomes of the five isolates were analyzed to identify genes encoding known pathogenicity factors (Table 3).

It was found that these isolates’ genomes included 18 of the 23 genes involved in adherence of *S. aureus* to cell wall, namely, *atl*, *clfA*, *clfB*, *ebpS*, *ebh*, *efb*, *fnbA*, *fnbB*, *eap/map*, *sasC*, *sasG*, *sraP*, *icaA*, *icaB*, *icaC*, *icaD*, *sdrC*, and *sdrD*. The genes *aap*, *bap*, *can*, *uafA*, and *sdrE* were not detected in these isolates.

Most of these genes were present in all five *S. aureus* genomes studied, particularly *atl*, *clfA*, *clfB*, *ebpS*, *ebh*, and *efb*, as well as *sasC* and *sasG*, which encode adherence factors anchored to the bacterial cell wall surface. In addition, all isolates possessed genes of the *ica* operon responsible for the synthesis of polysaccharide intercellular adhesin (PIA)—the key poly-β(1-6)-N-acetylglucosamine component of the biofilm matrix. As expected, the *bap* gene involved in initial attachment stages and the *ica*-independent pathway of biofilm maturation was absent in the studied genomes.

All five *S. aureus* isolates were found to possess the *sdrC* gene, whose product belongs to the family of microbial surface components recognizing adhesive matrix molecules (MSCRAMM). Interestingly, another gene encoding an MSCRAMM factor, *sdrD*, was identified in only isolate 17-21; furthermore, this isolate was also the only one to possess the *sraP* gene (also named *sasA*), which encodes a protein anchoring to the cell wall surface. Similarly, the *fnbB* gene was detected only in the genome of isolate 17-21, while *fnbA* was present in isolates 17-21 and 33-40. In contrast, the *eap/map* gene was found in all isolates except for 17-21.

### 3.5. Homology of Adherence Factor Genes

For comparative analysis, we selected the adhesin genes *clfA* and *ebpS*, which were represented in the genomes of all *S. aureus* strains studied. In many epidemiological studies, ClfA is considered the principal adherence factor.

**Figure 2:**
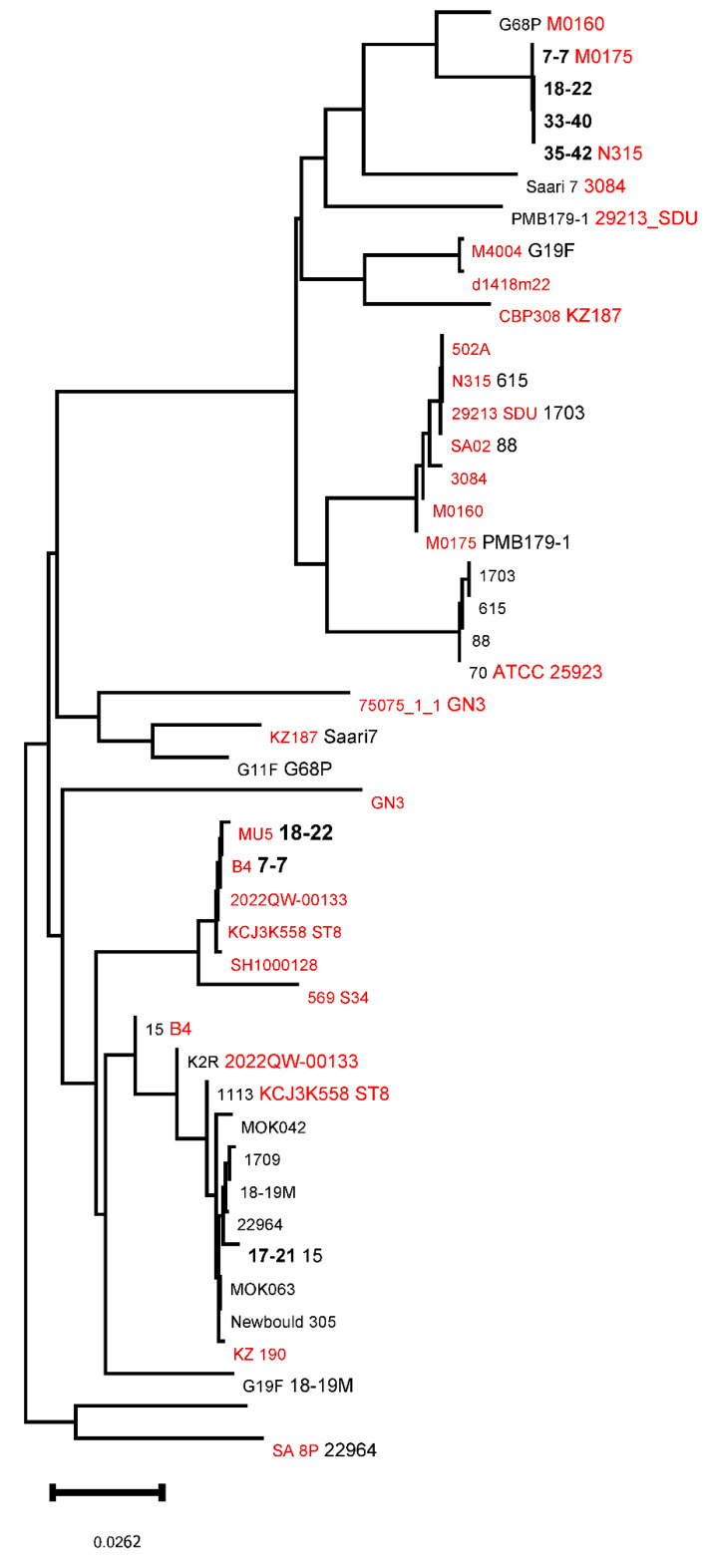
Comparative dendrogram of nucleotide sequencing of the gene *clfA* in *Staphylococcus aureus* strains isolated from human (red), bovine (black), and selected Yakut cow clones (bold black).

**Figure 3:**
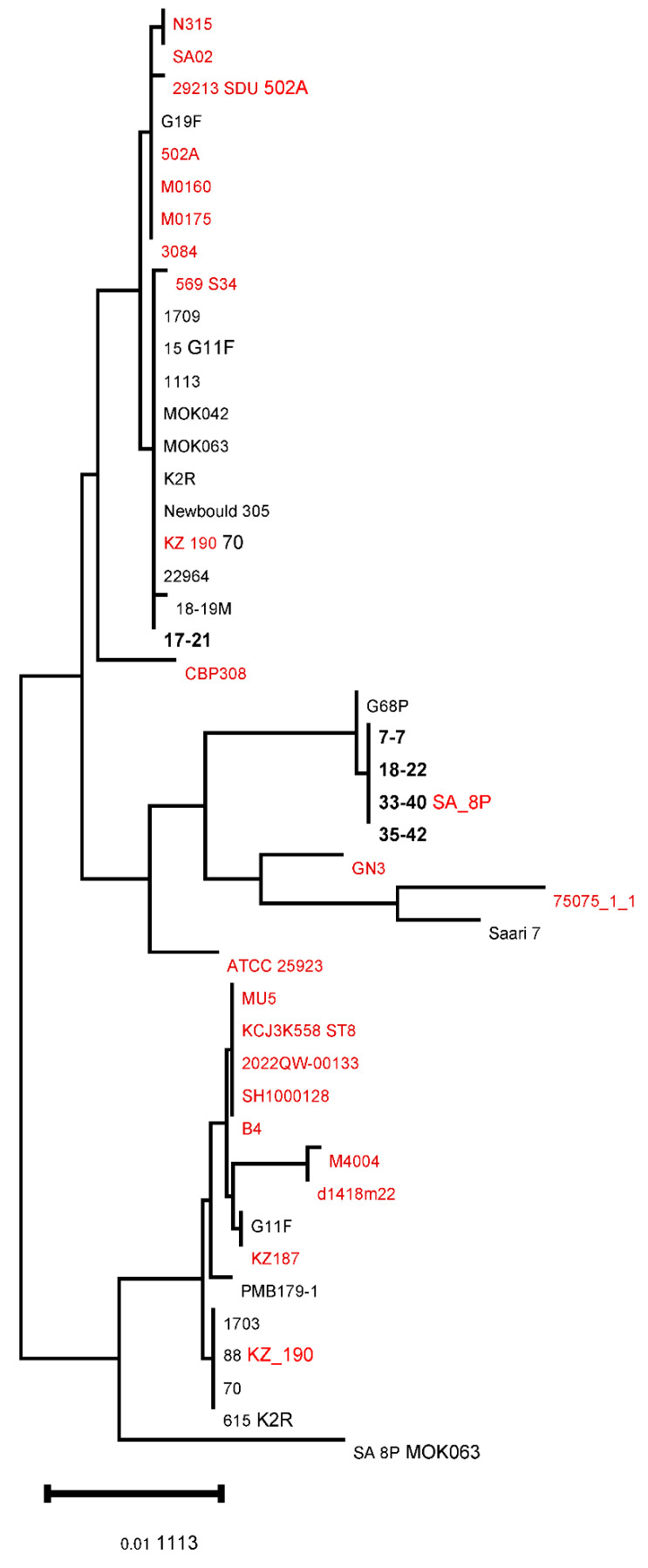
Comparative dendrogram of nucleotide sequencing of the gene *ebpS* in *Staphylococcus aureus* strains isolated from human (red), bovine (black), and selected Yakut cow clones (bold black).

As shown in the comparative dendrogram for *clfA* (Figure 2), four isolates (33-40, 35-42, 18-22, and 7-7) formed a cluster related to the strain G68P identified in cows. Similar results were obtained with the phylogenetic analysis of the *ebpS* gene (Figure 3). The isolate 17-21 was included in a separate clade for both genes.

### 3.6. Homology of α-Hemolysins

**Figure 4:**
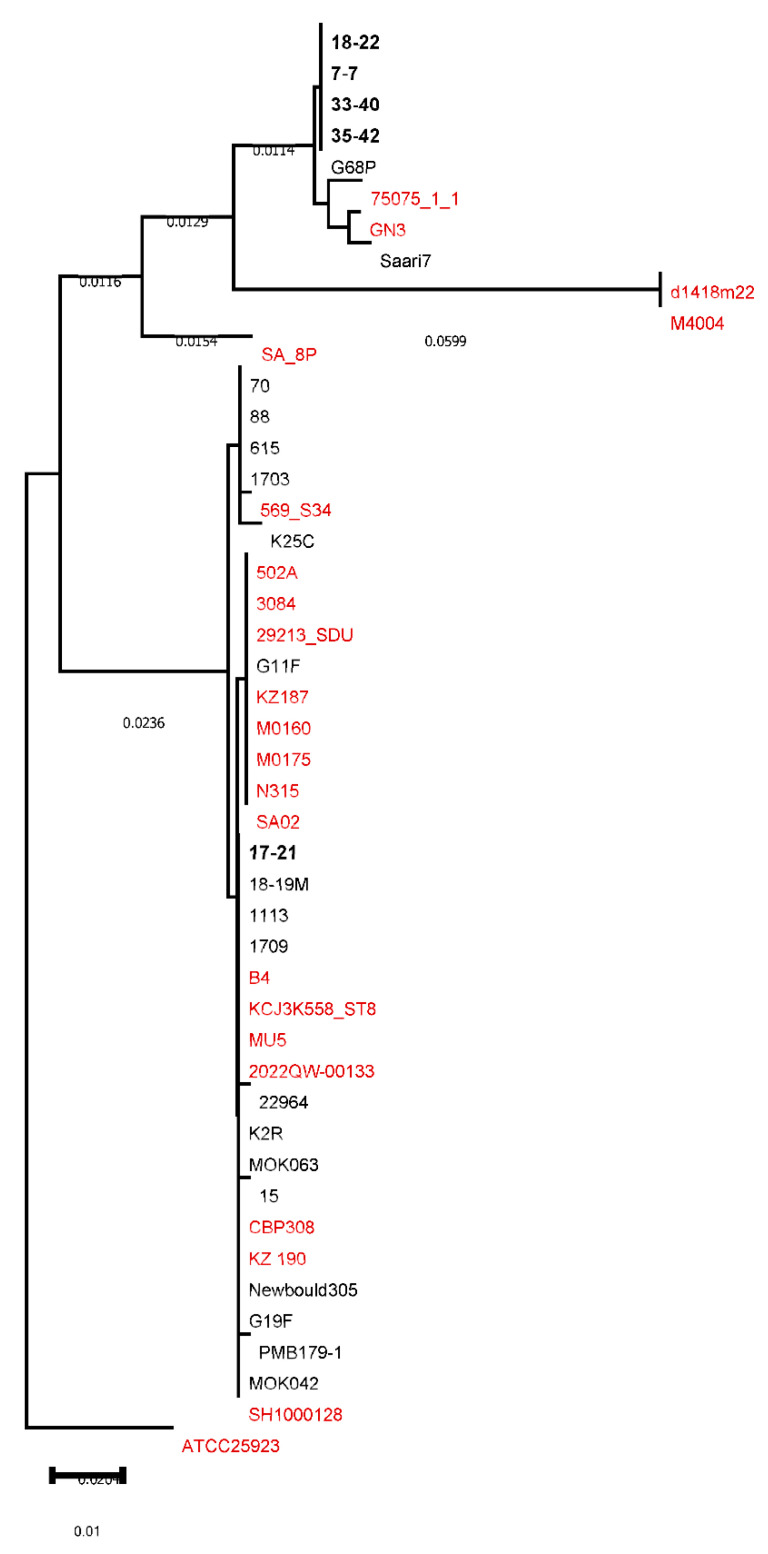
Comparative dendrogram of nucleotide sequencing of the gene *hla* in *Staphylococcus aureus* isolated from human (red), bovine (black), and selected Yakut cow clones (bold black).

The phylogenetic analysis of the *hla* gene (Figure 4) showed that four isolates (18-22, 7-7, 33-40, and 35-42) identified from Yakutian cows were separated into a clade with G68P, whereas in isolate 17-21, it formed a distant group with other isolates identified in other Russian cows.

### 3.7. Antimicrobial Resistance

At the genotype level, all five *S. aureus* isolates studied had similar antibiotic resistance profiles. All isolates were sensitive to the main antibiotics used to treat animals, namely methicillin, clindamycin, erythromycin, and sulfamedazole. At the same time, all isolates were resistant to antibiotics such as penicillin, tetracycline, ciprofloxacin, and gentamicin.

The phenotype analysis was conducted using the disc diffusion method; the results are presented in Table 4. All strains studied were found to exhibit multidrug resistance (MDR) according to the criteria summarized for *S. aureus* by Magiorakos et al. [22]. Isolates 17-21 and 35-42 were phenotypically resistant to four classes of antibiotics, and the other strains showed resistance to five or more classes of antimicrobial drugs. Regarding the number of antibiotics, all *S. aureus* isolates were resistant to at least five different compounds. For example, isolate 35-42 was resistant to gentamicin, rifampicin, ciprofloxacin, fusidic acid, and kanamycin, while isolate 17-21 was resistant to penicillin, gentamicin, kanamycin, rifampicin, and fusidic acid. Isolate 7-7 exhibited the broadest range of phenotypic resistance: it was resistant to all antibiotics tested except for clindamycin.

It is important to note that the five *S. aureus* isolates studied had different antibiotic resistance phenotypes, and their resistance profiles had no correlation with ST or CC. However, there were two antibiotics to which all five isolates were resistant, namely gentamicin and rifampicin. In addition, four of the five *S. aureus* strains were resistant to penicillin, ciprofloxacin, and kanamycin. On the other hand, only isolate 7-7 exhibited phenotypic resistance to tetracycline.

In the case of gentamicin, there was a correlation between genotype and resistance phenotype: all isolates were resistant to this antibiotic, a finding consistent with the presence of a gentamicin resistance sequence in their genomes. In contrast, a screening for tetracycline resistance phenotype showed the opposite result: only one strain, isolate 7-7 (sensitive only to clindamycin), was resistant to tetracycline. It is also interesting to note that all isolates possessed penicillin-resistant genotypes, while phenotypic resistance was recorded for all isolates except for 35-42. A contrasting situation was observed for clindamycin and erythromycin: the genotype analysis suggested that all isolates should be sensitive to these antibiotics, whereas phenotypically, isolates 18-22 and 33-40 were resistant to clindamycin, and isolates 7-7 and 18-22 were resistant to erythromycin.

The observed differences between phenotype and genotype were related not only to the presence of resistance genes acting directly (AMR-associated genes), such as the *bla* gene found in isolate 17-21 and the presence of *tet(38)* in all isolates, but also to the presence of various sets of efflux pumps. Particularly important factors are (i) the MepA efflux pump encoded by the chromosomal gene *mepA*, (ii) the multidrug export protein SAV1866, and (iii) the NorA multidrug efflux pump encoded by the *norA* gene. In 80% of isolates, *mepA* was present in two copies, and only isolate 17-21 possessed one copy of this gene. The genes for SAV1866 and NorA were present in 100% of isolates. Efflux pump regulators should also be considered. For instance, it is known that *mgrA*, which was found in all isolates in our study, encodes a transcription factor responsible for the regulation of *norA*, *norB*, *norC*, and *tet(38)* [23].

Genome search for antibiotic resistance genes identified a large set of genes potentially capable of neutralizing the effect of antibiotics on bacterial cells and protecting them from antibiotics (Table 4). All isolates contained genes encoding gyrase subunits, which restore the integrity and nativity of the genome under the damaging influence of quinolone antibiotics [24]. The gyrase A and gyrase B genes were present in the genomes of these isolates, at two copies each, and strain 33-40 possessed an additional copy of the gyrase A gene.

However, it should be noted that, despite the presence of AMR genes, the AMR phenotype of a given strain could differ from its genotype, suggesting that the presence of a gene in the genome does not necessarily imply protein production. For example, although all strains possessed *tet(38)*, only isolate 7-7 was tetracycline-resistant, while the others exhibited a discrepancy between phenotype and genotype and were sensitive to tetracycline. A similar case was observed for isolate 35-42, which possessed penicillin resistance genes but was nevertheless sensitive to this antibiotic.

### 3.8. Secretion of Alpha-Hemolysin

**Figure 5:**
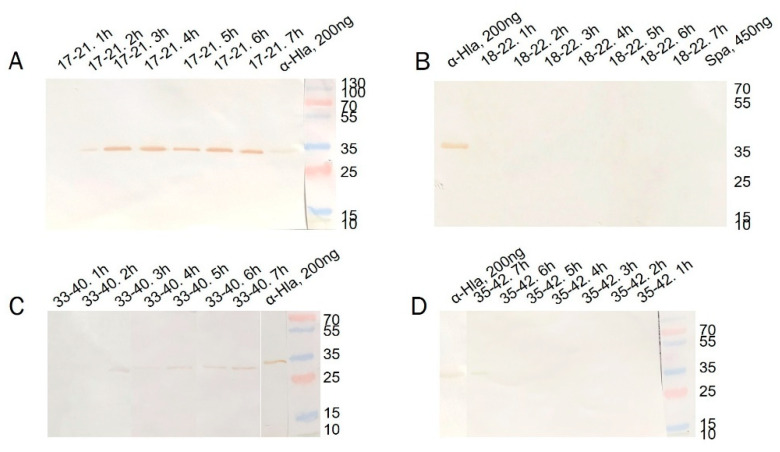
Western blot analysis of hemolysin production by *S. aureus* isolates identified from Yakutian cows. (**A**)—17-21; (**B**)—18-22; (**C**)—33-40; (**D**)—35-42; M—protein marker; positive control—purified hemolysin of *S. aureus*; negative control—protein A of *S. aureus* (SpA).

**Figure 6:**
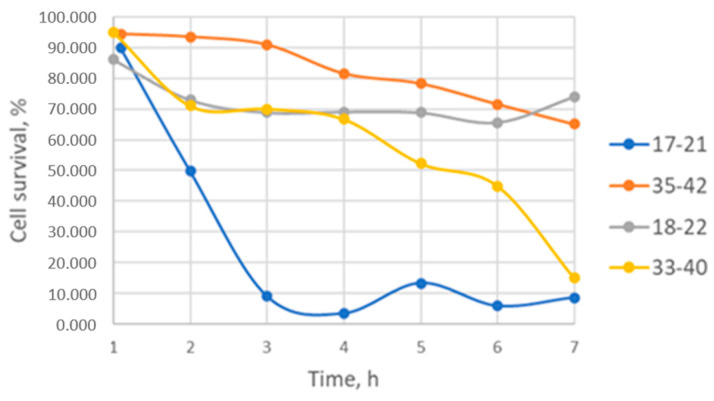
Hemolytic activity in culture liquid of five *S. aureus* isolates identified from Yakutian cows. The abscissa axis indicates the level of cell survival, in %. The ordinate axis denotes the time of culturing the strains, in h.

An analysis of hemolysin production also revealed phenotypic differences between the isolates, as demonstrated in both the Western blot analysis (Figure 5) and hemolysis assay with rabbit erythrocytes, considered to be the most sensitive method to evaluate the hemolytic activity of alpha-toxin [25] (Figure 6).

The presence of the protein itself was detected via immunoblotting. The use of two tests allowed for more accurate assessment of alpha-toxin production, given that microorganisms have multiple toxin genes in their genomes and might be capable of producing several pore-forming toxins (Table 2).

Strain 17-21 exhibited the highest hemolysin secretion, as detected both in the functional test and by immunoblotting. Strain 33-40 had significantly lower hemolytic activity. Strains 18-22 and 35-42 showed low lytic activity, and alpha-hemolysin was not detected by immunoblotting; it is probable that these isolates produce other toxins capable of lysing erythrocytes. Based on an evaluation of the protein standard (αHla) in the immunoblotting assay, the production of alpha-toxin by strains 17-21 and 33-40 amounted to 320 ± 37 and 100 ± 20 ng, respectively.

In sum, the results of the immunoblotting and hemolytic activity assays showed that, while all strains studied possessed the alpha-hemolysin gene, the corresponding protein was present in only some of the culture supernatants. These results indicate that the presence of a hemolysin gene in the genome of a *S. aureus* strain does not necessarily mean its production by the bacterial strain.

## 4. Discussion

Animals living in extreme environments can be carriers of unusual or even unique microorganisms. This article is devoted to the search for pathogenic microorganisms in Yakutian cows, in particular on *S. aureus*, as this pathogen is most associated with mastitis in cattle [26,27,28,29].

Since the milk samples were collected in a geographically remote region, with a low impact of external factors on the animals’ environment and keeping conditions, it became possible to identify the similarities and differences between the obtained *S. aureus* isolates and those isolated from cattle and humans in other regions. Using sequence typing, we investigated the specific characteristics of several strains within the same type; thus, clonal complexes could be combined based not only on the production of functional proteins but also on the potential host range. According to the results of the MLST analysis, the five *S. aureus* isolates were clustered into two distant STs, grouped into one CC and one singleton. Four isolates (7-7, 18-22, 33-40, and 35-42) were assigned to a singleton with ST1651 and a new spa-type t20403, followed by CC97 (ST97, t9303) for isolate 17-21. The involvement of CC97 in bovine mastitis has been globally distributed, including Algeria, Brazil, Canada, Chile, Germany, Ireland, Italy, Japan, Poland, Portugal, Rwanda, Switzerland, Tunisia, UK, and the United States [26]. A pangenome study conducted in 2020 noted that CC97 (as well as some other clonal complexes, such as CC8, CC1, CC20, CC9, CC7, CC5, and CC30) belonged mostly to the cluster associated with subclinical mastitis, although cases of clinical mastitis caused by CC97 members constituted 42% of all CC97-related cases [26]. In addition, there have been cases of CC97 transmission from cattle to humans. CC97 is traditionally considered a mastitis-associated strain in cattle, but cases of ST97 isolation from humans have also been reported; it is supposed that CC97 strains circulating in human populations are the result of transmission from livestock [28].

The widespread occurrence of the CC97 lineage indicates that these strains are adapted to their bovine hosts [29,30]. For instance, it was found that in *S. aureus* strains identified in bovine, genes involved in carbohydrate utilization are under diversifying selective pressure, which suggests adaptive evolution. In line with this genetic trait, bovine *S. aureus* strains were more efficient at utilizing lactose—the main source of carbohydrates in cow milk—than those obtained from humans or birds [22]. This observation is supported by an analysis of the SNP tree for *S. aureus* (Figure 1), where strain KZ_190 was found among the strains isolated from cows and belongs to CC97. However, its location in the CC97 branch is not accidental, as it was isolated from the breast milk of a patient with mastitis at a hospital.

It seems logical that, with some rare exceptions, strains of the same clonal complex are in their separate branches in all trees. Isolates 7-7, 18-22, 33-40, and 35-42 from Yakutian cows formed a separate branch together with strain G68P isolated in Switzerland and strain Saari7 isolated in Finland; based on the genomic characteristics, G68P is their closest evolutionary relative. It seems likely that isolates 7-7, 18-22, 33-40, and 35-42 are unique and endemic for Yakutian cattle, whereas isolate 17-21 might have been occasionally introduced from elsewhere. Considering that animals are kept in closed farm buildings at low ventilation levels for a long time during the winter period, it is likely that microbiota exchange occurs between cattle and farm personnel.

The fact that the *S. aureus* strains isolated from cattle and those isolated from humans are typically located in different tree branches suggests that staphylococci have, for the most part, been evolving independently in either niche, although their transfer from humans to cattle, and vice versa, is quite possible. However, successful transfer between humans and animals requires specific changes in adherence factor genes.

Comparative sequence analysis was performed on two *S. aureus* genes, *clfA* and *ebpS*, which encode adherence factors with a central role in attachment to host cells. These proteins have already been identified as factors involved in the pathogenesis of diseases caused by *S. aureus*. For instance, the N2N3 domain of ClfA can inhibit the C3b protein of the human complement system by binding to the regulatory factor H [31]. Moreover, while ClfA and EbpS occur rather commonly in *S. aureus* strains that infect humans or animals, it was reported that these factors may be exclusively associated with clinical mastitis [32]. In our study, the genes of these two proteins were found to be present in the genomes of all *S. aureus* strains.

Comparative phylogenetic analysis of the *clfA* and *ebpS* genes was conducted using *S. aureus* genomes isolated from human and bovine biological materials. For *clfA*, it was found that the gene sequence from strain 17-21 assigned to CC97 was closely related to the genes of the reference strains isolated from inflammation sites in both humans and cattle (Figure 2). At the same time, while the isolates in group ST1651 (7-7, 18-22, 33-40, and 35-42) also exhibited high affinity to either host, it can be seen from the chart that they belong to a different clade from the one containing strain 17-21.

Adherence factors are a group of proteins that facilitate the attachment of bacteria to the barrier systems of host cells and enable bacterial invasion in the body. The ability of bacteria to bind to the extracellular matrix or to host cells is a distinctive feature of *S. aureus* pathogenesis [33,34,35]. Furthermore, adherence factors participate in biofilm formation, promote invasion, contribute to defense from the host immune system, and facilitate the transition of the infection to a chronic state. The ability of bacterial pathogens to form biofilms is recognized as a central element of antibiotic resistance and has been shown to play a major role in persistent infections in animals and humans [36].

In our study, whole-genome sequencing identified numerous adherence-related factors that are characteristic of *S. aureus*. The isolates’ genomes were found to contain 18 genes involved in adherence, of which 13 (~72%) were present in all *S. aureus* strains studied. In particular, all isolates possessed all the genes of the *ica* operon responsible for the synthesis of key biofilm components—polysaccharide intercellular adhesins (PIAs). Interestingly, two other genes involved in biofilm formation, *aap* and *bap*, were not detected in any of the *S. aureus* isolates, although the product of *bap* is considered a specific biofilm protein in cattle-associated strains [37]. The presence of all *ica* genes and the absence of *aap* and *bap* indicate that these isolates form biofilms via the *ica*-dependent pathway, which aligns with the results of other studies [38,39,40].

The MSCRAMM family proteins play a key role in *S. aureus* adherence to host tissues. In our study, the isolates’ genomes were found to contain six genes encoding these proteins: *clfA*, *clfB*, *fnbA*, *fnbB*, *sdrC*, and *sdrD*. Among them, *clfA*, *clfB*, and *sdrC* were present in all five isolates, while the other genes occurred in only one or two isolates. Previous works have already reported the predominance of *clfA* and *clfB*, finding that these genes commonly occur in the majority of isolates [41,42]. In our work, the percentage of isolates possessing the *fnbB* gene was significantly lower than in previous studies. For instance, in a study in China, *fnbB* was a predominant gene along with *clfA* and *clfB* [43], while Polish researchers detected this gene in ~50% of isolates [44]. According to a study by Capra et al. [45], *fnbB* expression was associated with enhanced invasiveness of infection. ClfA has a vast range of functions that are not limited to interaction with barrier tissues. For instance, its coagulation with fibrinogen gamma chain allows *S. aureus* to evade the host’s immune response [46]. It also interacts with annexin-2 on epithelial cells in bovine mammary gland [47]. EbpS interacts with free elastin, whereas direct adherence to host tissue is mediated by ClfA [48]. A study by Pizauro et al. [49] noted the significance of EpbS, which was detected in 83.8% of isolates associated with clinical mastitis and in 68.8% of isolates in subclinical cases. This high occurrence is due to the fact that EpbS mediates the binding of bacteria to surface proteins or to soluble elastin of host cells; accordingly, this factor facilitates adherence to host cells at the initial stage of staphylococcal infection and is therefore of crucial importance [50].

Interestingly, some adherence factor genes exhibited a certain pattern of distribution in the genomes of the *S. aureus* isolates. In particular, *fnbB*, *sdrD*, and *sraP/sasA* were found in only isolate 17-21. On the other hand, the *eap/map* gene that encodes a multifunctional protein was detected in isolates 7-7, 18-22, 33-40, and 35-42, but not in 17-21. It has been shown that Eap/Map plays a central role in the adherence of *S. aureus* to eukaryotic cells and can modulate inflammatory response by interacting with ICAM-1 [51]. The distribution of the aforementioned genes in the isolates’ genomes suggests that they are ST-specific. Previously, this phenomenon was described for the same genes in a study by Naushad et al. [52].

Another factor that plays a significant role in pathogenesis is the activity of pore-forming toxins, particularly hemolysins [9]. Alpha-hemolysin causes the death of cells constituting surface barrier systems [53,54], enabling microorganisms to invade the mammary gland tissue. The involvement of alpha-hemolysin in the lysis of barrier-forming cells and the penetration of staphylococci into the underlying cell layers have also been demonstrated in other organisms, for example, the internalization of staphylococci in the human lung [55]. As a rule, internalization processes are accompanied by the activation of the host immune system [54,56,57].

All *S. aureus* isolates from Yakutian cows studied in this work contained a set of hemolysin genes (Table 2); among them, the most interesting one is alpha-hemolysin, which is the principal agent with the greatest impact on the disruption of the barrier systems and the penetration of the pathogen into the body.

The development of resistance to antimicrobial drugs is considered a virulence determinant, as antimicrobial resistance enhances pathogenicity in the host and facilitates transition to chronic infection [58,59]. In our study, 100% of the *S. aureus* isolates were resistant to at least four classes of antibiotics and were therefore characterized as showing multidrug resistance (MDR). In a previous study in western Russia, five of the eight *S. aureus* isolates tested for resistance to antimicrobial drugs (62.5%) were classified as MDR strains [60]. Although these statistics depend on the sample size, the results raise concerns about the hazards associated with MDR *S. aureus*. It is known that multidrug resistance complicates the treatment of infections, reduces the likelihood of successful treatment, and prolongs hospital stays, which worsens patient state and increases healthcare costs for both humans and animals [42].

Identification of antibiotic resistance genes is crucial for recognition and assessment of the pathogenic potential of *S. aureus* [61]. All isolates studied were shown to be resistant to at least five antibiotics used in veterinary practice. The antibiotic resistance genotypes of these isolates were also determined, and it was noted that the presence of sequences associated with antibiotic resistance did not always mean the presence of phenotypic resistance, and vice versa. For instance, phenotype screening showed resistance to beta-lactam antibiotics in 80% of isolates (n = 4), but at the genotype level, the *blaZ* gene responsible for resistance to this class of antibiotics was found in isolate 17-21 only. All other strains possessed the gene encoding the penicillin-binding protein, yet isolate 35-42 was sensitive to penicillin G.

All *S. aureus* isolates were shown to possess MDR efflux pumps. However, the activity of these elements needs to be enhanced to confer resistance. This fact can explain the differences between phenotype and genotype concerning, for example, sensitivity to tetracycline: although the *tet(38)* efflux pump gene was present in all isolates, only isolate 7-7 was resistant to tetracycline. The transcription factor MgrA plays an important role in the regulation of the activity of several membrane pumps; it interacts directly with the *norA* promoter and can indirectly influence the expression of *norB* and *tet(38)* [23,62]. Interestingly, the presence of genes encoding MDR efflux pumps, such as NorA and SAV1866, was associated with several virulence genes, as some virulence genes were found to be more common in isolates carrying these MDR efflux pumps. Additional studies can clarify some of the connections between these patterns. Nevertheless, whole-genome sequencing facilitates the screening of genomes for genetic determinants of antibiotic resistance, virulence genes, and their interactions.

The authors acknowledge that due to the very limited sample of isolates, the data cannot be generalized to the genetics of all staphylococci associated with mastitis found in Yakutia. However, the study reveals the new characteristics of five isolates from Yakutian cows, which can be used in future research for the characterization of new isolates associated with mastitis, contributing new data to the field of veterinary microbiology.

## 5. Conclusions

Five strains of *S. aureus* were isolated from the milk of Yakutian cows (Bos Taurus Turano-Mongolicus) that had previously been diagnosed with mastitis and received antibiotic treatment. The complete nucleotide sequences of their genomes were obtained using whole-genome sequencing methods. The five *S. aureus* isolates studied had different phenotypic antibiotic resistance profiles. In particular, all five isolates were resistant to gentamicin and rifampicin, and four of the five strains also showed resistance to penicillin, ciprofloxacin, and kanamycin. However, only one *S. aureus* strain, isolate 7-7, exhibited phenotypic resistance to tetracycline. The genes encoding two adherence factors, *clfA* and *ebpS*, were present in all strains and studied. Genome analysis showed that all strains possessed the *hla* gene. However, the expression of alpha-hemolysin varied between the strains. Phylogenetic analysis of these staphylococcus strains suggested that their evolution occurred independently in their respective ecological niches, although their transfer from cattle to humans, and vice versa, is possible. Isolates 7-7, 18-22, 33-40, and 35-42 are most typical to Yakutian cattle, while isolate 17-21 might have been introduced from a different region.

## Figures and Tables

**Table 1:** Strains used in this work and sequence types.

Source	Strain	GenBank Acc. No.	Sequencing Type	CC	Geographic Location
Cow	7-7	CP126626	1651		Russia, Yakutia
Cow	15	CP094925	4552	97	Mexico
Cow	17-21	CP126627	97	97	Russia, Yakutia
Cow	18-19M	NZ_WIQA00000000	97	97	Russia, Kirov
Cow	18-22	CP126630	1651		Russia, Yakutia
Cow	33-40	CP126631	1651		Russia, Yakutia
Cow	35-42	CP126629	1651		Russia, Yakutia
Cow	70	NZ_WIPL00000000	20		Russia, Tula
Cow	88	NZ_WIPN00000000	20		Russia, Tula
Human	502A	CP007454	5	5	USA, New York
Human	569_S34	NZ_JAKYYJ000000000	1	1	China
Cow	615	NZ_WIPP01000000	20		Russia, Perm
Cow	1113	PIOE01000000	7900	97	Russia, Central
Cow	1703	NZ_WOUL01000000	20		Russia, Moscow
Cow	1709	NZ_WNKR01000000	97	97	Russia, Saratov
Human	2022QW-00133	CP117203	8	8	USA, Michigan
Human	3084	NZ_JAQFVZ010000000	5	5	Russia, Nizhny Novgorod
Cow	22964	NZ_JAEADM010000000	97	97	Italy
Human	29213_SDU	NZ_JAQRFX010000000	5	5	Denmark
Human	75075_1_1	NZ_JAEAFG010000000	1162		Italy
Human	ATCC 25923	CP009361	243	30	USA, Seattle
Human	B4	NZ_JAHXKE000000000	8	8	Germany, Heidelberg
Human	CBP308	NZ_JAPVNO010000000	6	5	Italy, Cesena
Human	d1418m22	NZ_JAPDDY000000000	22	22	India
Cow	G11F	NZ_SZYL00000000	7		Switzerland
Cow	G19F	NZ_SZYN00000000	27	8	Switzerland
Cow	G68P	NZ_SZYR00000000	479		Switzerland
Human	GN3	AP017891	50		Japan
Cow	K2R	NZ_SZYS00000000	352	97	Switzerland
Human	KCJ3K533_ST8	NZ_JAMJGR000000000	8	8	USA, Florida
Human	KZ_190	NZ_JAKXMJ010000000	97	97	Russia, Kazan
Human	KZ187	NZ_JAIUGC000000000	7		Russia, Kazan
Human	M0160	KK003927	5	5	USA, Boston
Human	M0175	KK004599	105	5	USA, Boston
Human	M4004	NZ_OGBG00000000	22	22	Denmark
Cow	MOK042	CP029627	71	97	Ireland, Cork
Cow	MOK063	CP029629	97	97	Ireland, Tipperary
Human	MU5	CTYF00000000	239	8	Turkey, Istanbul
Human	N315	BA000018	5	5	Japan
Cow	Newbould 305	AKYW00000000	115	97	Canada, Ontario
Cow	PMB179-1	CP050690	15	15	South Korea
Human	SA_8P	NZ_JAMDXJ000000000	398		Algeria
Human	SA02	NZ_JAPKKA000000000	764	5	China, Shanghai
Cow	Saari 7	WOCT00000000	133		Finland
Human	SH1000128	JANFOA000000000	8	8	United Kingdom

**Table 2:** Pathogenicity factor genes detected in the genomes of five *S. aureus* isolates from Yakutian cows.

Gene	Isolate				
	7-7	18-22	33-40	35-42	17-21
Exotoxin	*seo*, *sem*, *sei*, *seu*, *sen*, *seg*	*seo*, *sem*, *sei*, *seu*, *sen*, *seg*	*seo*, *sem*, *sei*, *seu*, *sen*, *seg*	*seo*, *sem*, *sei*, *seu*, *sen*, *seg*	
Superantigen- like protein	*ssl1*, *ssl2*, *ssl3*, *ssl4*, *ssl5*, *ssl7*, *ssl8*, *ssl9*, *ssl10*, *ssl11*	*ssl1*, *ssl2*, *ssl3*, *ssl4*, *ssl5*, *ssl7*, *ssl8*, *ssl9*, *ssl10*, *ssl11*	*ssl1*, *ssl2*, *ssl3*, *ssl4*, *ssl5*, *ssl7*, *ssl8*, *ssl9*, *ssl10*, *ssl11*	*ssl1*, *ssl2*, *ssl3*, *ssl4*, *ssl5*, *ssl7*, *ssl8*, *ssl9*, *ssl10*, *ssl11*	*ssl1*, *ssl2*, *ssl3*, *ssl4*, *ssl5*, *ssl7*, *ssl8*, *ssl9*, *ssl10*, *ssl11*
Leukocydin *lukD/E*	*F/S*	*F/S*	*F/S*	*F/S*	*F/S*
Hemolysin	*hla*, *hlg*, *hld*	*hla*, *hlg*, *hld*	*hla*, *hlg*, *hld*	*hla*, *hlg*, *hld*	*hla*, *hlg*, *hld*

**Table 3:** Adhesin genes detected in the genomes of five *S. aureus* isolates from Yakutian cows.

Gene	Isolate				
	7-7	17-21	18-22	33-40	35-42
Accumulation-associated protein, *aap*	-	-	-	-	-
Biofilm-associated surface protein, bap	-	-	-	-	-
Autolysin, *atl*	+	+	+	+	+
Clumping factors, *clfA/B*	+/+	+/+	+/+	+/+	+/+
Elastin-binding protein, *ebpS*	+	+	+	+	+
Collagen adhesion protein, *cna*	-	-	-	-	-
Fibronectin-binding protein, *ebh*	+	+	+	+	+
*efb*	+	+	+	+	+
*uafA*	-	-	-	-	-
*fnbA/B*	-/-	+/+	-/-	+/-	-/-
Extracellular adherence protein, *eap*	+	-	+	+	+
Surface anchor proteins, *sasC/G/P*	+/+/-	+/+/+	+/+/-	+/+/-	+/+/-
Intracellular adhesion proteins, *icaA/B/C/D*	+/+/+/+	+/+/+/+	+/+/+/+	+/+/+/+	+/+/+/+
Ser-Asp-rich fibrinogen-binding protein, *sdrC/D/E*	+/-/-	+/+/-	+/-/-	+/-/-	+/-/-

**Table 4:** Antibiotic resistance of *S. aureus* isolates from Yakutian cows.

Isolate	Penicillin, 10 IU	Cefoxitin, 30 mkg	Ciprofloxacin, 5 mkg	Gentamicin, 10 mkg	Kanamycin, 30 mkg	Erythromycin, 15 mkg	Clindamycin, 2 mkg	Lincomycin, 15 mkg	Rifampicin, 5 mkg	Fucidin, 10 mkg	Tetraciclin, 30 mkg	Detected Resistance Gene
7-7	R	R	R	R	R	R	R	R	R	R	R	*mgrA*, *norA/B/C*, *pbp4*, *tet(38)*, *mepA*
17-21	R	S	R/S	R	R	S	R	S	R	R	R/S	*mgrA*, *norA/B/C*, *blaZ pbp4*, *tet(38)*, *mepA*
18-22	R	R	R	R	S	R	R	R	R	S	S	*mgrA*, *norA/B/C*, *pbp4*, *tet(38)*, *mepA*
33-40	R	S	R	R	R	S	R		R	S	NI	*mgrA*, *norA/B/C*, *pbp4*, *tet(38)*, *mepA*
35-42	S	S	R	R	R	S	S	S	R	R	R/S	*mgrA*, *norA/B/C*, *pbp4*, *tet(38)*, *mepA*

Note: R, resistant; S, susceptible; NI, none identified.

## Data Availability

The data presented in this study are available from the corresponding author upon request. All genomes have been deposited to GenBank, with accession numbers CP126626, CP126627, CP126629, CP126630, and CP126631.
